# Characterization of speech and language phenotype in the 8p23.1 syndrome

**DOI:** 10.1007/s00787-024-02448-0

**Published:** 2024-04-26

**Authors:** Çağdaş Karsan, Feyzanur Ocak, Talat Bulut

**Affiliations:** 1 Biruni University, Istanbul, Turkey; 2 Atlas University, Istanbul, Turkey; 3 Max Planck Institute for Psycholinguistics, Nijmegen, The Netherlands; 4 Istanbul Medipol University, Istanbul, Turkey

**Keywords:** 8p23.1 duplication syndrome, Speech, Language

## Abstract

The 8p23.1 duplication syndrome is a rare genetic condition with an estimated prevalence rate of 1 out of 58,000. Although the syndrome was associated with speech and language delays, a comprehensive assessment of speech and language functions has not been undertaken in this population. To address this issue, the present study reports rigorous speech and language, in addition to oral-facial and developmental, assessment of a 50-month-old Turkish-speaking boy who was diagnosed with the 8p23.1 duplication syndrome. Standardized tests of development, articulation and phonology, receptive and expressive language and a language sample analysis were administered to characterize speech and language skills in the patient. The language sample was obtained in an ecologically valid, free play and conversation context. The language sample was then analyzed and compared to a database of age-matched typically-developing children (*n* = 33) in terms of intelligibility, morphosyntax, semantics/vocabulary, discourse, verbal facility and percentage of errors at word and utterance levels. The results revealed mild to severe problems in articulation and phonology, receptive and expressive language skills, and morphosyntax (mean length of utterance in morphemes). Future research with larger sample sizes and employing detailed speech and language assessment is needed to delineate the speech and language profile in individuals with the 8p23.1 duplication syndrome, which will guide targeted speech and language interventions.

**Supplementary Information:**

The online version contains supplementary material available at 10.1007/s00787-024-02448-0.

## Introduction

The 8p23.1 duplication syndrome is a rare genetic condition that is estimated to occur in 1 out of 58,000 births [1]. Chromosome 8 is defined as an average chromosome by its length, gene content, repeat content and segmental duplication [2]. However, the distal part of its short arm exhibits some complex chromosomal abnormalities [1, 3–5]. The core 8p23.1 duplication interval contains 26 HUGO Gene Nomenclature Committee (HGNC) genes and five microRNAs [1]. This interval is positioned between the olfactory receptor/defensin repeats REPD (REPeat Distal) in distal 8p23.1 and REPP (REPeat Proximal) in proximal 8p23.1, which are thought to cause predisposition to reciprocal 8p23.1 deletions and duplications [1].

Duplications of 8p23.1 have been associated with a variable phenotype that includes somatic comorbidities (e.g., heart defects, ocular anomalies, balance problems, hypotonia, macrocephaly, and hydrocele), facial dysmorphisms (e.g., a broad forehead and thick eyebrows, and cleft lip and/or palate) and neuropsychiatric/behavioural symptoms (e.g., developmental delay, autism spectrum disorder, attention deficit hyperactivity disorder, and epilepsy) [3–8]. In addition, speech delay, learning difficulty and facial features resembling Kabuki syndrome were reported [9]. More detailed phenotypic features of 8p23.1 duplication cases reported in the literature are as follows:

- In one study, a summary of the phenotypic information obtained from 15 people using 12 postnatal probands is provided. Four of the postnatal probands had developmental delays, particularly in speech and language [1].
- One patient had a unilateral cleft lip, a complete cleft soft palate, and an incomplete cleft hard palate. In addition, echocardiogram examination showed ventricular septal defects (VSDs) and severe congenital stenosis of the aortic valve (AS) with a unicuspid aortic valve and congenital pulmonary valve stenosis [1].
- One patient had nipples that were slightly spread apart. He was observed to be developing socially and cognitively in line with his age, but his gross motor abilities were slightly behind his peers [1].
- One patient had developmental, cognitive, social, and language delay by the age of 5, speaking with only a few words. She was impulsive, had trouble falling asleep, and occasionally displayed oppositional behavior. She had a diagnosed attention deficit disorder [1].
- One patient was an outgoing, developmentally adequate girl with a large forehead, open, flat, and soft anterior fontanel, scant, fine white-blond hair, and very minimal occipital flattening. Although she had one erythematous salmon patch on the back of her head and hypopigmented eyes and skin with extremely light blue irises, she did not show any signs of freckling when exposed to sunlight. She suffered from sporadic nystagmus and was quite photophobic [1].
- Except for a few thin alopecia patches, one patient’s hair appeared normal and covered the majority of her head. She had significant colobomas on both of her irises as well as bilateral ptosis. She was surgically corrected for her large nasal bridge, well-repaired cleft lip, decreased cartilaginous tissue on both ears, and well-healed scars. In comparison to the right, the left ear was much less shaped. She had four fingers on her left hand with a medial cleft, four fingers on her right hand with leftover tissue from an additional digit surgically excised from the medial aspect of her right thumb, and extraordinary hands with differing numbers of digits. She had an extremely slender and tapered left foot with four toes and syndactyly between the second and third toes [4].

Although previous research associated the 8p23.1 duplication syndrome with speech and language delay [1, 5, 9, 10], these reports merely stated presence of developmental delay especially in the speech and language domain. Yet we have no information regarding which aspects of speech and language are affected in those patients and how severely.

To better understand the nature of speech and language issues encountered by individuals with this rare syndrome, it is necessary to carry out an in-depth assessment of their speech and language profiles. Therefore, the present study aimed to provide a detailed examination of speech and language, in addition to developmental and orofacial, features of a 4-year-old child who was diagnosed with 8p23.1 duplication syndrome. This is the first study reporting rigorous assessment of speech and language characteristics of a child with 8p23.1 duplication syndrome.

## Methods

Ethical approval was obtained for the present study from the Institutional Review Board of İstanbul Biruni University.

## Case

The patient is a 50-month-old boy who was diagnosed with the 8p23.1 duplication syndrome. He is a native Turkish speaker like his family members. They applied to Retorya Speech, Language and Development Center for speech therapy. After the initial interview, they were informed about the study and agreed to sign an informed consent form.

The patient’s parents are consanguineous. The mother stated that after an in vitro fertilization treatment, the baby was born healthy in due time and with about 2.49 kg birth weight. There is no other 8p23.1 duplication syndrome diagnosis in the family.

When the patient was 1.5 months old, the family applied to an ophthalmologist due to droopy eyelids and was referred to the neurology department, which, in turn, referred the patient to the genetics department in another hospital. When he was 9 months old, his genetic analysis was completed and the diagnosis of 8p23.1 duplication syndrome was verified. A molecular karyotyping test with a microarray system revealed a growth of 1.698 kbp in the 8p23.1 zone matching the critical area for 8p23.1 duplication syndrome. Also, the patient had undergone a genetic assessment for Down syndrome, which yielded a negative result. No additional exome sequencing was performed.

When he was seven months old, he started physiotherapy and walked at 16 months as reported by his mother. She also reported that he did not engage in any gestures or babbling during his infancy, but he said /bʌbʌ/ (Turkish word for daddy or papa) when he was six months old, and he used his first two-word utterance when he was two years old.

At the time of data collection, the patient was being followed up by the child neurology, genetics and ophthalmology departments. Also, his family described the patient as stubborn but talkative, not liking to share his toys with others, having weak hand muscles and not being able to jump. The patient attended a kindergarten from 08.00 to 17.30 every day except weekends.

## Data collection tools

### Oral-facial examination

An oral-facial examination form was used to inspect the oral-facial region for structural and functional integrity. This form is a valuable tool to detect phenotypic features of different syndromes as well as understanding the underlying causes of various articulation errors and problems with resonance. It includes evaluation of the size and symmetry of structures, caregiver reports or observation of reduced sensation (e.g., drooling, texture preferences), imitation of nonspeech and speech movement, and diadochokinetic rate for the production of syllabic sequences.

## Ankara developmental screening inventory (AGTE)

AGTE is used to evaluate children’s general development [11]. AGTE evaluates gross motor, fine motor, language-cognition, social and self-care skills and general developmental status of 0-6-year-old children. The inventory comprises 154 questions, to which parents or caregivers respond with a “yes”, “no” or “I don’t know”. The language-cognition subtest assesses the use of simple sounds and verbal behaviors, complex linguistic expressions, language comprehension and production, simple problem-solving skills, and comprehension of numeric and temporal concepts.

## Articulation and phonology test (SST)

SST is a standardized test for identification and evaluation of articulation disorders, and phonological delay/disorders in children aged between 2;0 and 7;11 [12]. It has the following three subtests.

### Articulation Screening Subtest (SET)

This subtest involves naming of 93 pictures depicting nouns, intended to evaluate Turkish consonants at different syllable and word positions. It also evaluates seven consonant clusters used in Turkish. Please note that Turkish has very few consonant clusters.

### Auditory discrimination (İAT)

İAT comprises 24 pages and 48 items to tests the ability to discriminate phonemes by visual and acoustic cues. There are two illustrations on each page which differ only by a single phoneme (minimal pairs). On each page, the clinician asks the patient to show the correct illustration by saying one of their names and repeating it six times.

### Phonological analysis Subtest (SAT)

SAT comprises 13 different theme pictures which are designed to elicit the use of certain words that are then used in phonological analysis. It is a semi-structured language sampling test to observe whether children use phonemes during natural speech in accordance with the phonological rules of Turkish.

## Turkish early language development test (TEDİL)

TEDİL is an adaptation of the Test of Early Language Development (TELD-3) [13]. TEDİL is a normative test used to evaluate receptive and expressive language skills of children between the ages of 2;0 and 7;11 [14]. It consists of two parallel forms (A and B) having 76 items each. We used Form A for this study, which has 24 items for semantics and 13 items for morphology/syntax in the receptive language domain, and 22 items for semantics and 17 items for morphology/syntax in the expressive language domain.

## Language sample analysis

One of the most ecologically valid sources of information regarding the child’s language performance is based on the observation and analysis of language samples acquired in a natural context while, for instance, the child is engaged in free play or conversation [15, 16]. For a speech and language pathologist, language samples are indispensable for several reasons. Results of a language sample analysis can be compared with a standardized test for a more in-depth understanding of the child’s needs. Language sample analyses can also help set therapy goals and monitor the treatment progress.

One of the prominent computerized language sample analysis tools is the Systematic Analysis of Language Transcripts (SALT). SALT is a computer program that analyzes children’s language samples in terms of morphology, syntax, semantics and discourse and allows comparisons with a normative database embedded in the program [17]. It is widely used to examine expressive language skills from 2;6 to 9;6 years of age. In this study, we used the Turkish Research Version 18 of SALT developed by Acarlar, Miller and Johnston [18], available on this website: https://dilornegianalizi.com. The Turkish database contains language samples from 321 typically developing children, as evaluated by the Denver Developmental Screening Test, aged between 2;6–9;6 years. The patient’s language sample was compared with samples from 33 age-matched speakers from the database as explained in the Results section. The procedure for language sample analysis involved taking a language sample, transcribing and coding it into the program, analyzing the patient’s language sample and comparing it with the database.

In this study, a language sample of 25 min was taken as a video recording by a researcher in the context of free play and conversation in one of the therapy rooms of Retorya Speech Language and Development Center. The first five minutes of the recording was treated as a warm-up period and, therefore, discarded. One researcher transcribed the acquired sample by watching the video while another researcher resolved any ambiguities in the transcription process (e.g. unintelligible utterances). The transcription was then coded into the computer program in accordance with the SALT conventions. Specifically, the transcription was divided into utterances, and bound morphemes were separated. Also, error codes were used in case of errors at morpheme, word or utterance levels. Other variables coded into the program include incomplete and unintelligible utterances, and mazes such as false starts, filled pauses and self-repetitions. This coding process was carried out by a researcher who was trained on SALT coding procedures. As with transcription, the SALT coding process was supervised by another researcher and any issues were resolved. Following the transcription and coding steps, the patient’s language sample was analyzed in terms of intelligibility (percentage of intelligible words and utterances), morphosyntax (mean length of utterance in words and morphemes), semantics/vocabulary size (total number of words, number of different words), discourse (percentage of responded questions, average speaker turn length in words and utterances), verbal facility (percentage of total number of words with mazes) and percentage of errors (percentage of utterances with errors). The patient’s scores were then compared with the database. The comparisons were based on the same number of complete and intelligible utterances made by the patient and the database to prevent potential confounds due to differences in utterance length.

## Results

### Oral-facial examination results

Examination of teeth revealed malocclusion (Class II), spaced arrangement and presence of cavities. Reduced range and weak strength of motion was observed while the patient puckered his lips. As for evaluation of the tongue, the frenulum was short. We observed incomplete excursion, reduced range of motion and reduced strength while the patient moved the tongue tip to the right. Reduced strength was also seen when the patient moved the tongue tip to the left. When examining rapid side-to-side movements, we observed that the rate of motion was reduced, and that the range of motion was reduced on the right. Evaluation of the hard and soft palates revealed a high and narrow arch. Further observations include right ptosis, downward slanting palpebral fissures, a broad nasal bridge, a short neck, posteriorly rotated ears, a folded auricle and low set, mild 2–3 cutaneous toe syndactyly, and over-sensitivity to touch.

### Developmental screening results (AGTE)

**Fig. 1:**
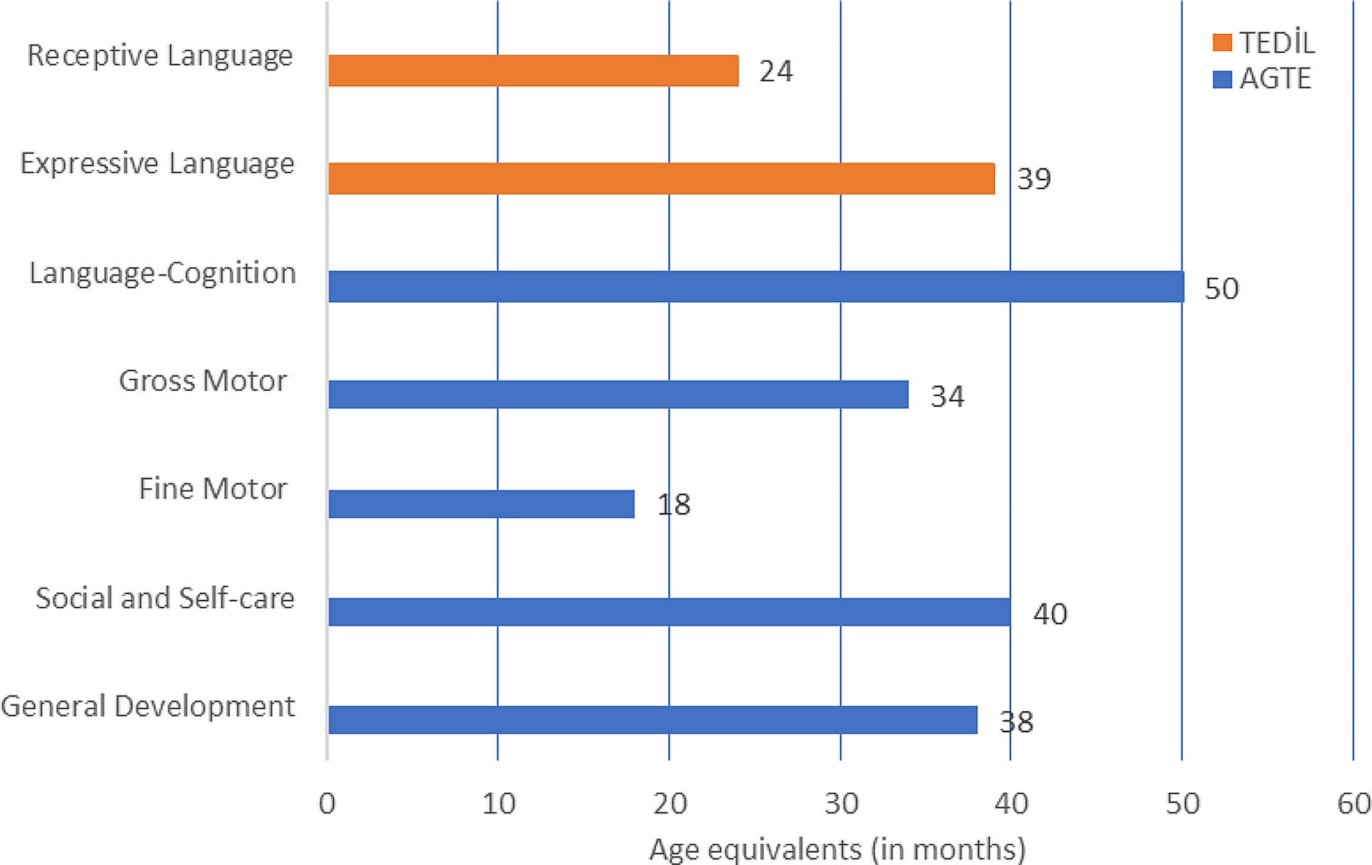
Patient’s age equivalents in language and other domains of development

Based on the AGTE raw results, the patient scored 54 (out of 65) in language-cognition, 16 (out of 26) in fine motor, 23 (out of 24) in gross motor and 35 (out of 39) in social and self-care domains. Thus, the patient’s general development raw score was 128 (out of 154), which was between 1 and 2 SD below the norm average. The patient’s age equivalents for general development and the developmental domains are summarized in Fig. 1. The patient scored lower than what would be expected of his age (50 months) in all developmental domains except for the language-cognition domain, where he was comparable to his peers. The developmental domain most at risk was fine motor, followed by gross motor. Overall, the patient’s age equivalent for general development was between 38 and 39 months. Based on these results, it was concluded that the patient’s development was at risk and required follow-up.

### Articulation and phonological assessment results (SST)

The patient’s scores from SET, İAT and SAT are summarized in Table 1. As shown in the table, the patient scored lower than what would be expected of his age in all three subtests of articulation and phonology. In particular, the patient was below the 1st percentile in İAT, pointing to a serious limitation in auditory discrimination of phonemes. The patient’s age equivalent was calculated as 3 ≤ for this subset.

According to the SET and SAT results, the patient performed the following phonological processes: Consonant deletion ([kibɾit] → [kibit]), frication ([bagaʒ] → [vaɡaʒ]), stopping ([jɯlan] → [dɯdan]), gliding ([ɾadjo] → [jadjo]), context-sensitive voicing/devoicing ([tabak] → [dabak], [zil] → [sil]), nasalization ([matʃ] → mʌn) and affrication ([ʃuɾup] → [tʃuɾup]). A phonemic inventory analysis revealed that all phonemes were present in the patient’s inventory except that the phoneme /z/ was absent at syllable initial and final positions. Sufficient context could not be obtained for the /h/ and /ʒ/ phonemes.

**Table 1:** Patient’s articulation and phonology test results

Test	Raw scores	Standard scores	Percentiles	Age equivalent
SET	11	89	23	4;5
İAT	56	2	< 1	≤ 3
SAT	13	86	17	4;2

### Language assessment results (TEDİL)

The patient obtained a standard score of 67 (out of 150), corresponding to a percentile of < 1, from the receptive language subtest of TEDİL, and a standard score of 85 (out of 150), corresponding to the 16th percentile, from the expressive language subtest. The patient’s TEDİL Z-score was − 1.93. The patient’s age equivalents for the receptive and expressive language subtests of TEDİL are illustrated in Fig. 1. These results suggest that the patient was at risk in terms of overall language development, but more so for the receptive language component.

### Language sample analysis results (SALT)

The patient produced 167 utterances using a total of 449 words. His language sample was compared with samples from 33 age-matched speakers from the database. These database samples were within 4 months of the patient’s age. The patient’s sample was compared with the database samples matched in length by the same number of utterances (*n* = 127 complete and intelligible utterances). All measures were interpreted using a standard deviation interval of 1.00 SD.

The patient’s intelligibility was within the normal range of 1 SD with 98.78% intelligible utterances and 99.52% intelligible words compared to the database samples (intelligible utterances *M* = 97.34, *SD* = 2.92, intelligible words *M* = 98.96, *SD* = 1.16). In terms of morphosyntax, the patient’s mean length of utterance (MLU) in words was 2.41, which was within the normal range compared to his database peers (*M* = 2.64, *SD* = 0.29). His MLU in morphemes was 3.91, which was 1.42 SD below the database mean of 4.70 (*SD* = 0.56). As for semantics/vocabulary, the patient used 164 different words (NDW) within an analysis set of 306 total number of words (NTW). NDW was 1.64 SD above the database mean of 140.85 (*SD* = 14.16), suggesting a relative strength in vocabulary diversity. In terms of discourse, the patient responded to 85.19% of questions asked by the clinician, which was within the normal range compared to the database mean of 89.53% (*SD* = 8.48). The patient produced an average of 1.38 utterances and 3.29 words per speaking turn, which was within normal range compared to database means of 1.44 utterances (*SD* = 0.22) and 3.78 words (*SD* = 0.99). As for verbal facility, of the patient’s NTW, 5.85% involved mazes including filled pauses, false starts, repetitions, or reformulations, which was within the normal range (database *M* = 6.33, *SD* = 3.94). Finally, 5.51% of the patient’s utterances contained errors at word and utterance levels, which was more than 3 SD higher than the database mean (*M* = 1.07, *SD* = 1.0). The patient made 1 error at the morpheme level (omission of a bound morpheme), 4 errors at the word level (use of a neologism or an error in word choice), and 2 errors at the utterance level (not possible to understand what is meant with the utterance given the context).

In summary, the language sample analysis results suggest that compared to his typically developing peers, the patient had lower MLU in morphemes and made more errors, while he did not exhibit risks in terms of vocabulary or discourse.

## Discussion

The 8p23.1 syndrome has been found to be phenotypically related with speech delay, autism and learning difficulties [5], although systematic and comprehensive assessment of speech and language skills have not been performed in this population. This is a first study attempt to analyze and describe speech and language-related characteristics of a case of 8p23.1 duplication syndrome. To that end, we conducted various speech and language evaluations using standardized tests and language sample analysis in addition to oral-facial and developmental examinations.

The oral-facial evaluation revealed facial abnormalities including right ptosis, downward slanting of palpebral fissures, posteriorly rotated ears and short neck. In addition, oral features including Class II malocclusion with weak lip sealing, short frenulum, narrow palate and decreased range of motion in tongue were present in the patient. These observations are consistent with earlier investigations of this population which reported a range of mild dysmorphic features [1, 3–6].

Developmental screening of the patient using AGTE revealed lower performance than what would be expected of his age (50 months) in all developmental domains except for the language-cognition domain, where he was comparable to his peers. The most compromised developmental domain was fine motor, followed by gross motor, and social and self-care. Given that the patient’s age equivalent for general development was between 38 and 39 months (between 1 and 2 SD below the norm average), the patient’s development was assessed to be at risk.

Although AGTE pointed to comparable language-cognition performance between our patient and the norms, further rigorous language assessment showed otherwise. Using SST, we detected a serious problem in auditory discrimination of phonemes. The other subtests of SST, on the other hand, yielded a mild disorder in articulation and phonology.

In addition to articulation and phonology, the patient’s performance in other language subdomains including semantics and morphology/syntax and functions receptive and expressive skills was assessed using TEDİL and SALT. Language assessment using TEDİL revealed serious delays in overall language development. This delay was greater for the receptive language component with an age equivalent of 24 months and a percentile of < 1 compared to the expressive language component with an age equivalent of 39 months corresponding to the 16th percentile. As reported in previous research [19], in certain cases receptive language can be impaired as much as expressive language with syndromic populations. However, we do not know why this discrepancy between the receptive and expressive language components occurred in our patient, and it remains to be shown by future research whether this would generalize to other individuals with the syndrome.

Observation and analysis of language samples acquired in a natural context constitutes one of the most ecologically valid sources of information regarding the child’s language performance [15, 16, 20]. Therefore, we took a language sample of the patient during free play and conversation, which was then transcribed and coded into SALT [17, 18, 21]. The sample was analyzed and compared to a database of age-matched typically-developing, Turkish-speaking children (*n* = 33) in terms of intelligibility, morphosyntax, semantics/vocabulary size/diversity, discourse, verbal facility and percentage of errors at word and utterance levels. When interpreted using a standard deviation interval of 1.00 SD, the only areas in which risks were identified were MLU in morphemes and percentage of errors. In particular, the patient’s MLU in morphemes was 1.42 SD below the database mean, and he made word- and utterance-level errors more than 3 SD higher than the database mean. MLU, particularly in morphemes, has been viewed as an important measure of language, particularly syntax, development [22–24]. Overall, the patient performed worse than his typically-developing peers in MLU in morphemes, but similarly in MLU in words and discourse, and even 1.6 SD above the database mean in the number of different words reflecting semantics/vocabulary size. These findings suggest that the patient was at a risk particularly in morphosyntax, while he did not exhibit risks in terms of vocabulary or discourse. The structural properties of the Turkish language may have influenced the present findings. In other words, Turkish is a morphologically rich language with the great majority of words containing more than one syllable being morphologically complex [25]. As a consequence, linguistic utterances usually involve bound morphemes on nouns and verbs serving grammatical functions. Therefore, being particularly at risk for morphosyntactic development, the patient in the present study may have presented with poor MLU in morphemes while showing MLU in words comparable to his peers. Similar morphological difficulties involving MLU in morphemes and morphological errors were observed in Turkish-acquiring children with various developmental disorders compared to their typically developing peers [26, 27], and were attributed to greater difficulty in grammatical features that have higher cognitive processing costs [26].

## Conclusion

The current study carried out a comprehensive speech and language assessment, in addition to oral-facial and developmental evaluation, of a single case with the 8p23.1 duplication syndrome. In line with previous research reporting descriptive and qualitative summary of speech and language development in this population, the present findings associated this syndrome with delays in certain speech and language functions [1, 5, 9, 10]. In particular, norm-referenced assessments revealed problems in articulation and phonology, receptive and expressive language skills, and morphosyntax.

These findings suggest that individuals with the 8p23.1 duplication syndrome should be carefully evaluated for speech and language functions. There may be delays or risks concerning speech and language performance and development in this population, which require early intervention by a speech and language pathologist. This study highlights the need for more robust speech and language assessment in this and similar genetic variants, ideally with greater sample sizes, to better delineate the compromised language subdomains. Detailed phenotyping of syndromic cases regarding language functions can potentially yield valuable information to aid patient-oriented and comprehensive therapy interventions as well as prognostic counseling.

## Electronic supplementary material

Below is the link to the electronic supplementary material.

Supplementary Material 1
